# The Cost of Resource Use Relative to the Development of the Antimicrobial Stewardship Program in a Tertiary Cancer Setting in Qatar

**DOI:** 10.3390/antibiotics14121204

**Published:** 2025-12-01

**Authors:** Dina Abushanab, Diala Alhaj Moustafa, Anas Hamad, Bhagyasree Adampally Sankar, Ziad G. Nasr, Hussam Alsoub, Daoud Al-Badriyeh

**Affiliations:** 1 Drug Information Center, Department of Pharmacy, Hamad Medical Corporation, Doha P.O. Box 3050, Qatar; 2 College of Pharmacy, QU Health, Qatar University, Doha P.O. Box 2713, Qatar; da1601696@qu.edu.qa (D.A.M.); ahamad6@hamad.qa (A.H.); znasr@qu.edu.qa (Z.G.N.); 3 Department of Pharmacy, National Center for Cancer Care & Research, Hamad Medical Corporation, Doha P.O. Box 3050, Qatar; 4 Department of Pharmacy, Ambulatory Care Center, Hamad Medical Corporation, Doha P.O. Box 3050, Qatar; bsankar1@hamad.qa; 5 Department of Medicine, Hamad Medical Corporation, Doha P.O. Box 3488, Qatar; halsoub@hamad.qa

**Keywords:** antimicrobial, stewardship, hematology, oncology, cost, resource use, cost saving, cost avoidance

## Abstract

**Background:** Infection is a typical consequence of cancer treatment due to its immunosuppressive nature, where the high use of antimicrobials raises the risk of antimicrobial resistance (AMR). The primary objective of an antimicrobial stewardship program (ASP) is to optimize antimicrobial use, reduce the emergence of AMR, and ensure cost containment. This study sought to assess the difference in cost of resource use with the ASP in the specialized hematology/oncology setting in Qatar, before and after ASP maturity. **Methods:** From the perspective of the public healthcare hospital, the research investigated the difference in the cost of resource use between the developed ASP and the preliminary ASP at the National Center for Cancer Care and Research (NCCCR), Qatar. The preliminary ASP was defined as the 12 months following the establishment of the ASP (i.e., May 2015 to April 2016), while the developed ASP was defined as the last 12 months of a 5-year ASP implementation (i.e., February 2019 to January 2020). Patient records were retrospectively reviewed. The overall difference in cost of resource use was based on cost savings, cost avoidance, and operational cost measures. **Results:** A total of 186 patients were included in the study, with 81 in the preliminary ASP and 105 in the developed ASP. While total resource utilization costs rose by 17% in the developed ASP, per-patient analysis revealed lower resource costs of Qatari Riyal (QAR) 1390 (USD 381) compared to QAR 1546 (USD 423) in the preliminary period. The developed ASP achieved reductions in antimicrobial consumption (−55.9%) and costs (−80.9%), along with a total cost avoidance of QAR 11,969,651 (USD 3,288,366). Overall, the program resulted in a net annual reduction of QAR 13,205,840 (USD 3,618,038), which equates to QAR 180,910 (USD 49,564) saved per patient. **Conclusions:** At the NCCCR, Qatar, it seems that running the ASP for five years, with presumed development in its practices, was associated with reductions in antimicrobial costs, operational expenses, and overall resource spending.

## 1. Introduction

Despite the fact that antimicrobials have been very beneficial to human health, the inappropriate and unregulated use of these agents in clinical and agricultural settings could soon undermine their efficacy primarily due to the emergence of antimicrobial resistance (AMR) [1], including the collateral damage to the normal gut flora, which leads to increased risk of antibiotic-resistant bacteria, such as Clostridioides difficile infections (CDIs) [2]. The inappropriate prescription of antimicrobials by hospital physicians was estimated to constitute as high as half of all patient cases in some settings [3]. In cancer settings, specifically, infection is a typical consequence of cancer treatment due to its immunosuppressive nature, and the required high use of antimicrobials raises the risk of AMR and its morbidity and mortality consequences [4].

AMR not only has devastating effects on an individual level but also results in a substantial economic burden at the larger scale of the system [5], constituting added spending due to increased use of medications and screening tests, increased readmissions, and prolonged hospital stays [6]. The World Health Organization (WHO), for instance, estimates that by 2050, AMR could result in the deaths of 10 million people [6], which is projected to incur a global economic cost ranging between United States dollars (USD) 300 billion and USD 1 trillion [5].

To promote the rational use of antimicrobials, regulatory bodies have implemented antimicrobial stewardship programs (ASPs). The key objectives of ASPs are to reduce healthcare costs, curb antibiotic overuse, and combat antimicrobial resistance [7]. Talking about antibiotics, as an example, implementing an ASP alongside other strategies that curtail irrational drug use could potentially save up to 1.6 million lives and USD 4.8 billion by 2050 across the 33 member nations of the Organization for Economic Co-operation and Development (OECD) [8]. The benefit of implementing ASP strategies in oncology and hematology settings is particularly evident in the literature, including reduced drug use, improved clinical outcomes, and decreased associated costs [9,10].

In Qatar, the ASP was implemented in 2015 by the public secondary/tertiary healthcare provider, i.e., Hamad Medical Corporation (HMC), with the aim of promoting the rational use of antimicrobials. The ASP at HMC focused on 18 antimicrobials chosen based on their spectrum of activity, inappropriate usage, and cost.

The running of ASP strategies can incur significant financial expenses and, consequently, it is natural for hospital administrations to look to ensure that such investments will lead to comparable cost savings. In HMC, adhering to best international practices, the question around ASP is not whether it is a practice to implement, but whether reduced spending on resource use with the ASP practices has been maintained since ASP inception in 2015. Within this context, the current research sought to estimate the difference in the economic value of resource use with the ASP in Qatar between implementation and after development, i.e., after 5 years of implementation, focusing on the adult oncology and hematology medical settings at the National Center for Cancer Care and Research (NCCCR), one of the major specialized hospitals of HMC [11].

## 2. Results

### 2.1. Patient Demographics

A total of 186 patients were enrolled in the study: 81 during the preliminary ASP period and 105 during the developed ASP period. Patients in the developed ASP group were older, with a mean age of 54.7 ± 19.89 years, compared to 47.8 ± 17.62 years in the preliminary ASP group (*p* = 0.015). Gender exhibited statistical significance (*p* = 0.008), with men constituting the majority in the preliminary ASP group (66.7%), while women formed the majority in the developed ASP group (53.3%). There was a statistically significant disparity in the distribution of medication classes between the two groups (*p* < 0.001). Antifungals were more prevalent in the preliminary ASP group (91.4%), while antibacterials dominated in the developed ASP group (94.3%). Patients in the preliminary ASP group were more likely to have a severe Charlson Comorbidity Index (CCI) score compared to those in the developed ASP group (*p* < 0.001). Included cases comprised therapeutic and empiric antimicrobial use, while prophylactic antimicrobial therapy represented <2% of total cases and was managed under the ASP review process. Further demographic details are provided in Table 1.

### 2.2. Classification of Antimicrobials

Among the initial antimicrobials administered in patients, fluconazole (n = 47, 57.3%) followed by caspofungin (n = 12, 14.63%) were the predominant choices during the preliminary ASP period. In contrast, the developed ASP period featured the top two antimicrobials ertapenem and ciprofloxacin, accounting for 32.38% and 29.5%, respectively. Subsequent antimicrobial administration was mostly in the preliminary ASP period, in 30 versus 6 patients, with voriconazole (n = 6, 24%) and posaconazole (n = 5, 20%) commonly used during that period. Additional information about the administered antimicrobials is provided in Supplementary S1.

### 2.3. Economic Analysis

#### 2.3.1. Cost Savings with Antimicrobial Consumption

The developed ASP was associated with a substantial reduction in antimicrobial consumption (defined daily dose (DDD)) of 55.9%, and in the associated cost, 80.9%. The developed ASP period incurred a total antimicrobial cost of QAR 300,321 (USD 82,506), whereas the preliminary ASP period incurred QAR 1,569,699 (USD 431,236), which is a positive total cost saving of QAR 1,269,378 (USD 348,730) in favor of the developed ASP. On a per-patient basis, the developed ASP incurred a cost of QAR 2860 (USD 784) per patient, compared to QAR 19,379 (USD 5309) in the preliminary phase, resulting in a per-patient cost saving of QAR 16,519 (USD 4526).

Further details on the total cost of the DDD for antimicrobials during the study periods are outlined in Supplementary S2.

#### 2.3.2. Cost Savings with Resource Utilization

Total resource utilization costs were higher by approximately 17% during the developed ASP period, amounting to QAR 145,956 (USD 40,098), compared to QAR 125,245 (USD 34,408) during the preliminary ASP period, which is a negative total cost saving of QAR 20,711 (USD 5689) in favor of the preliminary ASP. However, when adjusted on a per-patient basis, the developed ASP period demonstrated lower resource costs of QAR 1390 (USD 381) compared to QAR 1546 (USD 423) in the preliminary period, corresponding to a per-patient positive cost saving of QAR 156 (USD 43) in favor of the developed ASP. A breakdown of resource utilization in both periods is given in Table 2.

#### 2.3.3. Cost Avoidance

The total cost of resources avoided during the developed ASP period was QAR 5,950,155 (USD 1,634,658), in contrast to QAR 17,919,806 (USD 4,923,024) during the preliminary ASP period, yielding a positive total cost avoidance of QAR 11,969,651 (USD 3,288,366) in favor of the developed ASP. When analyzed on a per-patient basis, the developed ASP period also showed a lower cost of QAR 56,668 (USD 15,526) per patient, in contrast to QAR 221,232 (USD 60,612) per patient in the preliminary period, representing a per-patient cost avoidance of QAR 164,564 (USD 45,086). Details of cost avoidance expenses in both periods are available in Table 2.

#### 2.3.4. Operational Cost

The developed ASP incurred lower total operating costs than the preliminary ASP, QAR 61,714 (USD 16,954) versus QAR 74,190 (USD 20,382), resulting in a positive total cost saving of QAR 12,477 (USD 3428) in favor of the developed stage. On a per-patient basis, the developed ASP incurred costs of QAR 588 (USD 161) per patient, compared to QAR 916 (USD 251) in the preliminary phase, resulting in a per-patient positive cost saving of QAR 329 (USD 90).

#### 2.3.5. Overall Difference in Cost of Resource Use

The developed ASP was associated with a net annual reduced resource use of QAR 13,205,840 (USD 3,618,038), corresponding to QAR 180,910 (USD 49,564) per patient relative to the preliminary ASP. The detailed outcomes of the cost–benefit analysis are provided in Table 3.

### 2.4. Sensitivity Analysis

#### 2.4.1. One-Way Sensitivity Analysis

The study’s outcome demonstrated insensitivity to all input uncertainties at both total and per-patient levels. Results, input uncertainties, and sampling distributions of the one-way sensitivity analysis are presented in Supplementary S3 and S4.

#### 2.4.2. Multivariate Sensitivity Analysis

Multivariate sensitivity analysis indicated a 100% probability that the developed ASP is associated with lower resource use on the total outcomes (Supplementary S5). Comprehensive details of the multivariate sensitivity analysis, input uncertainties, and sampling distributions at both total and per-patient levels are found in Supplementary S3.

**Figure 1:**
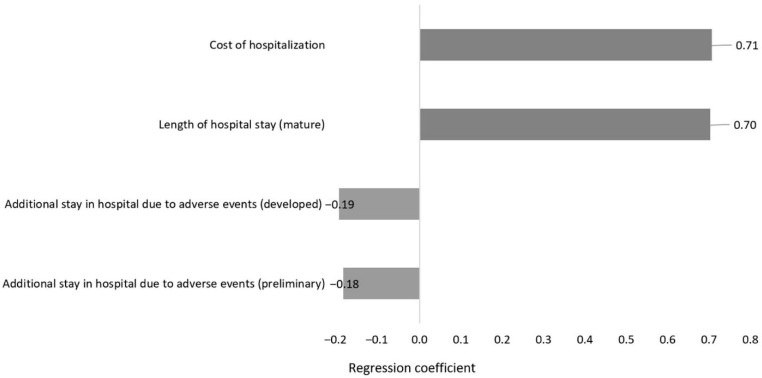
Effects of variables on cost of resource use reduction in favor of developed ASP.

Ranking the analysis inputs as per influence on the study total outcome, a regression Tornado analysis revealed the predominant influence of hospitalization costs on the study outcome, whereas the additional hospital stay due to adverse drug events (ADEs) exerted the least impact on the outcome (Figure 1).

### 2.5. Secondary Outcomes (Clinical Outcomes)

The length of hospital stay during initial disposition was significantly shorter in the developed ASP period compared to the preliminary ASP period, with approximately 7 days versus 34 days, respectively (*p* = <0.001). Hospital readmissions were not reported in either group. Furthermore, a lower incidence of CDI was observed in the developed ASP group compared to the preliminary ASP group (15.2% vs. 30.9%, respectively; *p* < 0.001). No significant differences were observed between the two groups in terms of infection-related death, infection resistance, and ADEs. Further details regarding the clinical outcomes are provided in Table 4.

## 3. Discussion

The findings of this retrospective study shed light on changes in the cost of resource use between the developed ASP and the preliminary ASP at the only specialized cancer hospital in Qatar, comprehensively accounting for the different aspects of resource use in healthcare [12,13]. The international studies and their results are generally not comparable to our results because, in this study, we report the cost of spending with the developed use of ASP compared to its use during the preliminary period. Comparing pre-ASP and post-ASP implementation was unfeasible since HMC introduced electronic medical records in 2015, the same year that ASP was implemented.

Overall, the developed ASP phase showed reductions in both total and per-patient healthcare spending. The program achieved a net annual saving of QAR 13,205,840 (USD 3,618,038), which translates to QAR 180,910 (USD 49,564) saved per patient compared to the preliminary phase. This decrease in overall expenditure was largely attributed to a 55.9% reduction in antimicrobial consumption (DDD) and an 80.9% decline in associated drug costs, resulting in total savings of QAR 1,269,378 (USD 348,730), or QAR 16,519 (USD 4526) per patient. Additionally, there was a positive cost avoidance of QAR 11,969,651 (USD 3,288,366) overall, which equated to QAR 164,564 (USD 45,086) per patient, reflecting fewer infection-related complications, reduced hospitalizations by 27 days, and a decrease of nine CDI cases. Operational costs were also reduced by QAR 12,477 (USD 3428) overall, or QAR 329 (USD 90) per patient, indicating improved efficiency in stewardship team activities despite the expanded program scope. While total resource utilization costs were 17% higher due to increased diagnostic and laboratory activity, by QAR 20,711 (USD 5689), a per-patient analysis revealed a cost saving of QAR 156 (USD 43) in favor of the developed ASP, reflecting optimized diagnostic targeting and enhanced patient throughput.

It is important to note that while the one-way and multivariate uncertainties confirmed the study outcome against uncertainties and variability in study inputs, it is impossible to assume that this decreased spending with the developed ASP relative to the preliminary ASP is due to enhanced effectiveness with the developed ASP, especially given the limitations of increased CCI score with the preliminary ASP and differences in the antibiotic versus antifungal use between groups, in addition to other potential confounding factors, such as the potential antimicrobial shortage, the subsequent conservation efforts on antimicrobial use, and the several environmental factors that can affect antimicrobial resistance, but are not necessarily related to the antimicrobial consumption. Indeed, while with cumulative experience, one would assume that the ASP practice in HMC becomes more effective, all that we can conclude from this study is that there is a decrease in the cost associated with the developed ASP, without necessarily associating this with increased developed ASP performance.

The notable decrease in antifungal use observed during the developed ASP period aligns closely with the program’s strategic focus on diagnostic stewardship and antifungal restriction policies introduced after 2016. In the early years of ASP implementation, antifungal therapy, especially fluconazole, caspofungin, and voriconazole, was often initiated empirically for cancer patients with febrile neutropenia or unexplained fever, frequently before microbiological confirmation. This approach was precautionary in an immunocompromised population but led to excessive antifungal consumption and costs. With the advancement of the ASP, several interventions significantly altered this trend. First, antifungal approval processes were established, requiring authorization from an ID physician or ASP pharmacist before starting high-cost antifungal treatments. Second, the integration of fungal biomarkers and rapid culture diagnostics enhanced the ability to rule out invasive fungal infections sooner, thereby reducing unnecessary antifungal continuation. Third, clinical decision support tools within the electronic medical record (Cerner^®^) offered real-time prescribing guidance and automatic alerts for prolonged therapies. Additionally, regular educational sessions and cancer stewardship rounds encouraged evidence-based escalation and timely de-escalation of broad-spectrum or antifungal agents.

Consequently, antifungal prescriptions transitioned from empirical to targeted use, while optimized antibacterial stewardship practices (including IV-to-oral switch protocols, adherence to local antibiograms, and narrowing of spectrum) became more prominent. This systematic evolution accounts for the proportional increase in antibacterial use and the notable decrease in antifungal utilization observed during the advanced ASP phase. Importantly, antifungal agents, especially echinocandins and triazoles, are significantly more costly and necessitate longer treatment durations compared to antibacterial agents like ertapenem or ciprofloxacin [14]. Consequently, this shift in the antimicrobial mix likely contributed to the 80.9% reduction in antimicrobial consumption costs and shorter hospital stays. Overall, these programmatic enhancements not only optimized therapy but also yielded significant economic benefits by lowering drug acquisition costs, reducing hospital length of stay, and minimizing infection-related complications such as CDI. In any case, however, regardless of the effect of ASP development in HMC on the reduction in cost of resource use, the observed decline in antimicrobial consumption costs during the developed ASP period is in line with the existing literature emphasizing the effectiveness of ASPs in optimizing antimicrobial use and reducing unnecessary prescriptions. In one study, in Canada, the implementation of an ASP reduced the cost of antimicrobial use by 16.6% in leukemia patients [10]. In another study, decreasing the consumption of a single drug, vancomycin, by 18%, after the implementation of an ASP, resulted in cost savings of over USD 173,000 [15].

Antimicrobial use often serves as a proxy outcome measure for national-level policies, with the belief that reduced use would lead to lower resistance rates and better future clinical and economic outcomes. Our study indicated that the decrease in DDDs, particularly in the antifungal agents, accounted for the substantially lower spending during the developed ASP stage. This can be attributed to the NCCCR’s adherence to restrictive prescribing privileges for these agents. Additionally, the intentional effort to de-escalate and discontinue such agents might explain HMC’s success in reducing antimicrobial consumption. A cross-sectional multi-center study raised concerns by revealing that 7.5% of patients received antifungal treatment despite lacking documented invasive candida infections [16]. The literature consistently supports the impact of ASP interventions on antifungal use, resulting in enhanced performance measures [17].

Conversely, the increased resource utilization costs observed during the developed ASP period may be attributed to heightened awareness and surveillance practices in response to ASP development. Similar trends have been reported in the literature, suggesting that increased testing and monitoring may arise as a consequence of intensified ASP activities [18]. The economic trade-offs between increased resource utilization costs and the associated benefits need to be carefully considered in ASP evaluation and implementation.

Cost avoidance was considered to estimate the economic value of prevented outcomes, including avoided readmissions, reduced hospitalization duration, and prevention of ADEs and CDI. This method, consistent with the previous ASP literature [19,20,21], reflects the monetary value of resources conserved due to improved antimicrobial management when direct observation is not feasible.

The pronounced cost avoidance during the developed ASP period underscores the value of comprehensive ASPs in mitigating the economic burden of avoidable complications. Although there were no studies in cancer setting specifically investigating cost avoidance associated with ASPs, our finding is consistent with studies in non-cancer settings highlighting that ASPs contribute to reducing healthcare-associated infections, readmissions, and ADEs [16,17,18,19,20,21,22,23,24,25]. These results reflect the broader social impact of ASPs in terms of cost savings and enhanced patient safety.

The shorter length of hospital stays observed during the developed ASP period aligns with the literature evidence that ASPs expedite patient recovery by optimizing therapy and avoiding unnecessary hospitalization [26]. Similarly, the reduced incidence of CDI during the developed ASP period in our study mirrors the literature evidence of the role of ASPs in reducing the risk of nosocomial infections and improving infection control [7,27].

While our multivariate sensitivity analysis showed that the developed ASP was associated with less resource spending in 100% of iterations, it also demonstrated via the Tornado analysis that this lesser spending, to a substantial extent, is driven by the hospital bed day cost. This is aligned with the literature evidence, where, for example, in Europe, the proportion of a bed day saved through the implementation of ASPs represents 60–80% of the total hospital stay, while in the US, the proportion of a bed day saved was lower (32%) [12]. A systematic review of studies conducted in the US reported shorter hospital stays and higher cost savings per patient following ASP implementation, and this was mainly due to the high cost of a hospital bed day in the US [12].

These findings not only guide national ASP strategies in Qatar but also enhance the global evidence base that supports the economic sustainability of antimicrobial stewardship programs in specialized cancer care. For the international audience, the non-HMC settings can benefit from this study in relation to recognizing the important aspects of resource use to monitor as they affect whether implementing an ASP is associated with increased or reduced spending over time, in addition to recognizing factors that contribute to the value of resource use the most in relevance to an ASP. Locally in HMC, we need to emphasize that all resource use aspects in the current study are site- and local practice-specific, and therefore future work should extend the setting and measure the resource cost change associated with the development of ASPs in other HMC clinical practice units such as cardiology and women’s health.

This study is not devoid of limitations. Inherent confounding and biases are intrinsic to the retrospective nature of the study design. While the long-term benefits of ASPs are important [28,29], our study lacks long-term data on ASP outcomes. Furthermore, the study’s economic analysis may not capture all relevant resources as only direct medical costs were factored into the analysis, potentially underestimating the positive impact of ASPs, primarily related to indirect costs (i.e., productivity loss).

Nevertheless, despite the potential uncertainties associated with these limitations, the sensitivity analysis results were consistent with those of the base-case analysis, which is reassuring and does confirm measured outcomes as robust.

As policymakers deliberate on healthcare strategies, supporting ASPs after implementation should be a priority, ensuring both immediate cost reductions and long-term sustainability in healthcare resource management. Here, ongoing educational initiatives conducted by the ASP team on infectious disease diagnosis and treatment based on approved national guidelines will likely play a pivotal role in maintaining minimized exposure to key unwarranted antimicrobials, as evident in the literature [23,24].

## 4. Methods

### 4.1. Study Design and ASP Description

This study employs a retrospective review of patient records who received at least one of the targeted antimicrobials at HMC during two distinct periods. The first period, referred to as “preliminary ASP”, spanned from the immediate implementation of ASP at HMC up to 12 months, which is from 1 May 2015 to 30 April 2016. The second period, denoted as “developed ASP”, encompassed the last 12 months of a 5-year ASP implementation at HMC, from 1 February 2019 to 31 January 2020. The required ethical approval was obtained from the Medical Research Center, HMC (MRC-01-20-213).

The ASP at HMC was formally initiated in April 2015, in alignment with the core elements set by the Infectious Diseases Society of America (IDSA) and the Society for Healthcare Epidemiology of America (SHEA) [30]. Initially, the program had a limited scope (“preliminary ASP”), consisting of a small team with one infectious disease physician and one clinical pharmacist who reviewed restricted antimicrobials daily and convened monthly to discuss prescribing trends. At this stage, stewardship interventions mainly relied on manual audits, feedback, prospective reviews of restricted antimicrobials, and clinician education on empirical antibiotic selection.

Over the next five years, the ASP grew in both capacity and sophistication (“developed ASP”). The team expanded to include a dedicated infectious disease consultant, a clinical microbiologist, an infection control practitioner, and a stewardship nurse. The developed ASP introduced structured antimicrobial approval processes, real-time electronic surveillance through the hospital’s electronic medical record system (Cerner^®^), automated alerts for duplicate or prolonged therapies, and periodic reports on antimicrobial use (DDD) and resistance trends to hospital administration. Educational outreach also increased, featuring regular feedback for prescribers and updates to guidelines. At the NCCCR, ASP activities were integrated into oncology and hematology care rounds, focusing on de-escalation, IV-to-oral conversion, and optimization of antimicrobial prescribing.

### 4.2. Study Setting

The study was conducted in NCCCR, the only specialized adult cancer hospital in Qatar. Part of HMC, NCCCR offers specialized care in hematology, oncology, critical care, and palliative care, featuring around 80 beds [11].

### 4.3. Study Population

The ASP at HMC targeted the optimization of 18 antimicrobial medications, namely, cefepime, linezolid, teicoplanin, tigecycline, ertapenem, amikacin, colistin, ciprofloxacin, moxifloxacin, aztreonam, ceftazidime, daptomycin, anidulafungin, fluconazole, amphotericin, caspofungin, posaconazole, and voriconazole.

Eligible patients were those admitted to and staying at NCCCR within the specified study periods, receiving any of the 18 targeted antimicrobials, starting antimicrobial treatment within 3 days of admission, and receiving the same antimicrobial for 48 consecutive hours. Critically ill patients were excluded from the study during the database query phase. This group was defined as those admitted to the Intensive Care Unit (ICU) at any time during their antimicrobial treatment, or those treated under the HMC critical care protocol. This definition aligns with institutional policies that dictate distinct antimicrobial decision pathways for ICU cases, which prioritize urgent stabilization and life-support management over stewardship oversight. The critical care protocol does not limit antimicrobial decision-making or focus on optimization, as urgency takes precedence. Excluding these patients was necessary to prevent confounding from protocol-driven, non-stewardship antimicrobial use and to ensure that the analysis accurately represented cases managed within the ASP framework.

### 4.4. Sample Size

Consistently with successful examples in the literature [31], this study’s population was duration-based and not a calculation-based sample size. Patient recruitment was conducted over two one-year periods: preliminary ASP use (1 May 2015 to 30 April 2016) and developed ASP use (1 February 2019 to 31 January 2020). Because cost-analysis studies are about making a cost estimation, and are not concerned with hypothesis testing like clinical research, even if a cost-analysis is underpowered, it still provides important information for guiding decision makers in healthcare systems [32,33,34].

### 4.5. Statistical Analysis

Patient data was tabulated, and IBM SPSS (Statistical Package for the Social Sciences) version 29 was utilized for analysis. Categorical variables were presented as numerical and percentage measurements, while continuous variables were expressed as mean and standard deviation. Because the two study periods are over four years apart, and to assess how comparable these periods are, we needed to assess the similarity of treated patients and whether there are considerable demographic shifts that may affect ASP outcomes. Continuous variables were evaluated for normality using the Shapiro–Wilk test. Student’s *t*-test was used for normally distributed data, and the Mann–Whitney U test for skewed distributions. Categorical variables were compared using the Chi-square test or Fisher’s exact test, as appropriate. A significance level of 5% (alpha value) was set.

### 4.6. Outcome Measures

#### 4.6.1. Primary Outcome

The change in the monetary value of resource use between the developed state of the ASP practices and the preliminary state of the ASP practices in NCCCR.

#### 4.6.2. Secondary Outcomes

- All-cause death within 30 days of hospitalization.
- Infection-related death, defined as a death that occurred within 30 days of hospitalization, where the primary or contributing cause was recorded as an infection in the electronic medical record (Cerner^®^), based on physician notes, microbiology results, or reviews by infectious disease consultants.
- Infection-related readmission, defined as any hospital readmission within 30 days of discharge with a documented infection diagnosis or a positive microbiological culture that required antimicrobial therapy.
- Occurrence of hospital-onset CDI.
- Duration of hospital stay due to infection-related reasons.
- ADEs associated with antimicrobials. ADEs were identified based on documented adverse reactions or clinical notes indicating toxicity linked to antimicrobial therapy, in line with definitions from the World Health Organization (WHO) and the National Coordinating Council for Medication Error Reporting and Prevention (NCC MERP) [35,36]. ADEs were recognized when a temporal relationship between antimicrobial administration and the onset of adverse clinical manifestations (such as rash, hepatotoxicity, nephrotoxicity, cytopenia, or gastrointestinal disturbances) was recorded in the patient’s electronic medical record (Cerner^®^) by the treating physician or the infectious disease consultant. Documentation sources included progress notes, medication-related alerts, and laboratory values indicating drug-related toxicity (e.g., elevated creatinine levels with nephrotoxic antibiotics). Events labeled as “suspected adverse drug reaction,” “toxicity,” or “treatment-related complication” were also classified as ADEs. Development of antimicrobial resistance during hospitalization.

All outcomes were extracted directly from the Cerner^®^ system based on documentation from the clinical teams. This study utilized existing clinical records, which reflected assessments that had already been conducted and validated by the infectious disease consultants and clinical pharmacists involved in the ASP workflow.

### 4.7. The Change in Monetary Value of Resource Use

The overall change in the monetary value of resource use between the developed and preliminary ASPs was measured based on cost savings, cost avoidance, and operational costs. Outcomes were analyzed and presented both as total monetary values and per-patient values to provide a comprehensive comparison of system-level and patient-level resource utilization between the two ASP phases.

#### 4.7.1. Cost Saving

- The reduction in therapy cost because of an assumed reduction in antimicrobial consumption with the developed ASP relative to the preliminary ASP was measured using DDD [37]. The DDD is defined as the dose of medication most commonly used for the most common indication in adults. An assumed reduction in the overall resource utilization cost with the developed ASP relative to the preliminary ASP was measured. The total financial cost of resources for each patient was calculated based on micro-costing, utilizing the unit cost of specific resources and their utilization frequency. Resource use of interest was related to reduced usage of diagnostic, laboratory, and culture tests, as well as a transition from intravenous (IV) to oral medication during the initial three days of antibiotic therapy.

#### 4.7.2. Cost Avoidance

Cost avoidance was computed as the avoided cost due to decreased hospital readmissions, shortened hospital stays, lower occurrences of CDIs, and fewer ADEs resulting from inappropriate antimicrobial use.

- The length of hospitalization and the hospital readmission of interest in the study were those deemed to be infection-related and/or relevant to the use of antimicrobials. The total cost of hospitalization length was determined by multiplying the daily cost of hospitalization by the number of hospitalization days for each patient.
- In line with the above, microeconomic analysis of individual-level resource utilization was also applied to calculate CDI costs. The overall cost of CDI was derived based on the unit cost of CDI diagnosis and care. Standard care for CDI consisted in vancomycin 125 mg orally four times daily for ten days, assuming all CDI patients underwent CDI cultures [38].
- Based on expert consensus in the current study and relevant to previous studies [39], the cost of an ADE was calculated on the conservative assumption that any injectable or non-injectable antimicrobials will lead to an additional 2 days or 1 day of hospital stay in the relevant unit, respectively.

#### 4.7.3. Operational Cost

The operational cost of ASP implementation constituted the operational expenses associated with running the ASP. These expenses encompassed the time spent by pharmacists and physicians in daily data collection, attending daily clinical rounds, and participating in monthly committee meetings.

- With the preliminary ASP, the program comprised the costs of one physician and one clinical pharmacist spending five hours in monthly committee meetings. Additionally, there are the costs of one clinical pharmacist and one physician spending three hours daily on data collection, as well as the cost of one physician spending one hour daily in clinical rounds.
- With the developed ASP, the program comprised the costs of an infectious disease consultant, a clinical pharmacist, a clinical microbiologist, an infection control practitioner, and a nurse spending ten hours monthly in meetings. Also included are the costs of one physician fellow and one clinical pharmacist spending three hours daily on data collection and one hour daily in clinical rounds.

### 4.8. Cost Data

Given the hospital perspective of the study, only direct medical costs were considered in the analysis. The unit costs of selected resources during patient admission were provided by HMC’s finance and costing section, while the pharmacy department provided the unit costs of antimicrobial medications. All costs were denoted in QAR and adjusted for the financial year 2023, using the Qatari Health Consumer Price Index if necessary. All costs were also presented in USD.

### 4.9. Sensitivity Analysis

Sensitivity analysis was employed to enhance the study’s robustness and generalizability of its findings, whereby the analysis was re-run after varying values of study inputs of interest within pre-defined ranges of value uncertainty.

- A one-way sensitivity analysis assessed the impact of uncertainty in specific inputs on study conclusions, where values of uncertain input variables were each substituted with new values, for an input variable at a time. Targeted inputs in the analysis were the cost of hospitalization, length of initial admission, and length of additional hospital stays due to ADEs, with each assigned a 20% uncertainty range.
- Multivariate sensitivity analysis was conducted by simultaneously targeting various underlying uncertain inputs, simulating real-life uncertainty. Here, the cost of hospitalization, length of initial admission, and additional hospital stays due to ADEs were of interest. Each input was varied within a ±10% uncertainty range.

Both one-way and probabilistic analyses use the Monte Carlo simulation approach, facilitated by @Risk-7.6 (Palisade Corporation, Ithaca, NY, USA). This method involved re-running the study analysis for 1000 iterations, randomly sampling, in each run, values from an assigned uncertainty range to each of the uncertain values of study inputs. The current study utilized a triangular-type distribution for random input selection.

## 5. Conclusions

This study illustrates that the advancement of the ASP practices at the NCCCR in Qatar was associated with significant and quantifiable economic advantages. Over a span of five years, the developed ASP phase was associated with a net annual spending reduction of QAR 13,205,840 (USD 3,618,038), which translates to about QAR 180,910 (USD 49,564) saved per patient. This was driven by decreased antimicrobial consumption, improved cost avoidance, and reduced operational costs. Although total resource utilization increased due to expanded diagnostic activities, per-patient analyses indicated lower overall costs, highlighting more efficient and targeted care. Clinically, these enhancements were associated with a 27-day shorter hospital stay and a decrease of nine cases of CDI, demonstrating both economic benefits and improvements in patient safety.

## Figures and Tables

**Table 1:** Patient demographics.

Variable	Preliminary ASP (n = 81)	Developed ASP (n = 105)	*p*-Value
*Gender, n (%)*			
Male	54 (66.7)	49 (46.7)	0.008
Female	27 (33.3)	56 (53.3)	
*Age, mean ± SD*	47.8 ± 17.62	54.7 ±19.89	0.015
*Weight, mean ± SD*	67.18 ± 23	73 ± 27.3	0.158
*Nationality, n (%)*			
Arab	48 (59.3)	77 (73.3)	0.115
Asian (non-Arab)	30 (37)	28 (26.7)	
Western	1 (1.2)	0 (0)	
African (non-Arab)	2 (2.5)	0 (0)	
*Allergy, n (%)*			
Yes	1 (1.2)	73 (29.3)	0.074
No	81(98.8)	176 (70.7)	
*Class of medications, n (%)*			
Antibacterial	7 (8.6)	99 (94.3)	<0.001
Antifungal	74 (91.4)	6 (5.7)	
*Number of medications received n (%)*			
1	56 (69.1)	101 (96.2)	<0.001
2	20 (24.7)	2 (1.9)	
3 or more	5 (6.2)	2 (1.9)	
*Location of infection, n (%)*			
Neck	1 (1.2)	0 (0)	<0.001
Skin and soft tissue	2 (2.5)	11 (10.5)	
Respiratory	4 (4.9)	2 (1.9)	
Gastrointestinal	3 (3.7)	9 (8.6)	
Abdomen	1 (1.2)	0 (0)	
Genitourinary	1 (1.2)	22 (43.1)	
Blood	36 (44.4)	36 (34.3)	
unknown	33 (40.7)	25 (23.8)	
*Charlson Comorbidity Index score, n (%)*			
0	0 (0)	36 (34.3)	<0.001
Mild	32 (39.5)	23 (21.9)	
Moderate	16 (19.8)	17 (16.2)	
Severe	33 (40.7)	29 (27.6)	

ASP: antimicrobial stewardship program.

**Table 2:** Resource utilization during the study ASP periods.

Resource	Preliminary ASP	Developed ASP
**Value of resource utilization, QAR (USD)**		
Culture performance before receiving therapy	640 (USD 18)	720 (USD 198)
Laboratory test performance before receiving therapy	24,730 (USD 6794)	28,892 (USD 7937)
Biopsy performance before receiving therapy	0	0
Culture performance after receiving therapy	2880 (USD 791)	2943 (USD 809)
Laboratory test performance after receiving therapy	28,994 (USD 7966)	37,641 (USD 10,341)
Biopsy performance after receiving therapy	0	0
Switching from IV to oral	68,001 (USD 18,682)	75,760 (USD 20,813)
**Total cost**	**125,245 (USD 34,408)**	**145,956 (USD 40,098)**
***Cost saving,*** *in favor of preliminary ASP*	***Negative 20,711 (USD 5689)***	
**Cost per patient**	**1546 (USD 423)**	**1390 (USD 381)**
***Cost saving,*** *in favor of developed ASP (per patient)*	***Positive 156 (USD 43)***	
**Cost avoidance (QAR, USD)**		
Hospitalization	16,827,888 (USD 4,623,046)	4,567,221 (USD 1,254,731)
Clostridium difficile infection and its management	5050 (USD 1387)	3536 (USD 988)
Rehospitalization within 30 days	0	0
Adverse drug events	1,086,868 (USD 298,590)	1,379,108 (USD 378,876)
**Total cost**	**17,919,806 (USD 4,923,023)**	**5,950,155 (USD 1,634,657)**
***Cost avoided*** *, in favor of developed ASP*	***Positive 11,969,651 (USD 3,288,365)***	
**Cost per patient**	***221,232 (USD 60,612)***	***56,668 (USD 15,526)***
***Cost avoided*** *, in favor of developed ASP (per patient)*	***Positive 164,564 (USD 45,086)***	

ASP: antimicrobial stewardship program; QAR: Qatari Riyal; USD: United States Dollar.

**Table 3:** Cost–benefit analysis due to the maturity of antimicrobial stewardship program.

Parameter	Total Value Difference in Favor of Developed ASP QAR (USD)	Value Difference in Favor of Developed ASP per Patient QAR (USD)
Cost saving in terms of DDDs	1,269,378 (USD 348,730)	16,519 (USD 4526)
Cost saving in terms of resource utilization	Negative 20,711 (USD 5689)	Positive 156 (USD 43)
Total cost saving	1,248,666 (USD 345,151)	16,675 (USD 4568)
Cost avoidance	11,969,651 (USD 3,288,366)	164,564 (USD 45,086)
Operational cost	Positive 12,477 (USD 3428)	Positive 329 (USD 90)
Net reduction in monetary spending	13,205,840 (USD 3,618,038)	180,910 (USD 49,564)

DDD: defined daily dose; ASP: antimicrobial stewardship program; QAR: Qatari Riyal; USD: United States Dollar.

**Table 4:** Clinical outcomes.

Variable	Preliminary ASP (n = 81)	Developed ASP (n = 105)	*p*-Value
Length of hospital stay (initial disposition), *mean ± SD*	34.02 ± 51.21	7.12 ± 10.47	<0.001
Readmission, *n (%)*			
Length of hospital stay (second disposition), *mean ± SD*	0	0	
All-cause death, *n (%)*			
Yes	1 (1.2)	7 (2.8)	0.46
No	80 (98.8)	242 (97.2)	
Infection-related death, *n (%)*			
Yes	0 (0)	0 (0)	1
No	81 (100)	105 (100)	
Infection resistance, *n (%)*			
Yes	0 (0)	0 (0)	1
No	81 (100)	105 (100)	
Clostridioides difficile, *n (%)*			
Yes	25 (30.9)	16 (15.2)	<0.001
No	56 (69.1)	89 (84.8)	
Adverse drug events, *n (%)*			
Yes	0 (0)	0 (0)	1
No	81 (100)	105 (100)	

ASP: antimicrobial stewardship program.

## Data Availability

The data presented in this study are available in the article and the Supplementary Materials.

## Supplementary Materials

The following supporting information can be downloaded at: https://www.mdpi.com/article/10.3390/antibiotics14121204/s1, Supplementary S1. Classification of antimicrobials. Supplementary S2. Defined daily doses during preliminary and developed use of ASP. Supplementary S3. Sensitivity analyses and their uncertainty distributions. Supplementary S4. Probability of reduction in cost of resource use in favor of developed ASP, one-way sensitivity analysis. Supplementary S5. Reduction in cost of resource use in favor of developed ASP, multivariate sensitivity analysis.
