# Supplementary material for: The Cost of Resource Use Relative to the Development of the Antimicrobial Stewardship Program in a Tertiary Cancer Setting in Qatar
Source: Antibiotics (Basel). 2025 Dec 1;14(12):1204. doi: 10.3390/antibiotics14121204 (PMC12729906; doi:10.3390/antibiotics14121204)

Supplementary S1. Classification of antimicrobials

Antimicrobial	Preliminary ASP, n=81	Developed ASP,n=105
First antimicrobial, n (%)		
Ciprofloxacin	1 (1.23)	31 (29.5)
Cefepime	0	4 (3.81)
Ertapenem	0	34 (32.38)
Moxifloxacin	1 (1.23)	20 (19.04)
Amikacin	4 (4.94)	3 (2.86)
Fluconazole	47 (58.02)	1 (0.95)
Linezolid	0	2 (1.9)
Tigecycline	1 (1.23)	2 (1.9)
Amphotericin	0	2 (1.9)
Anidulafungin	1 (1.23)	1 (0.95)
Caspofungin	12 (14.82)	2 (1.9)
Posaconazole	11 (13.58)	0
Cefazolin	0	1 (0.95)
Voriconazole	3 (3.7)	0 (0)
Teicoplanin	0 (0)	2 (1.9)
Total	***81 (100)***	***105 (100)***
Second antimicrobial, n (%)		
Ertapenem	1 (4)	0
Amphotericin	3 (12)	0
Amikacin	2 (8)	0
Voriconazole	6 (24)	0
cefazolin	0	1 (25)
Posaconazole	5 (20)	1 (25)
Fluconazol	3 (12)	1 (25)
Caspofungin	2 (8)	1 (25)
Anidulafungin	3 (12)	0 (0)
Total	***25 (100)***	***4 (100)***
Third antimicrobial, n (%)		
Ciprofloxacin	1 (20)	1 (50)
levofloxacin	1 (20)	1 (50)
Tigecycline	1 (20)	0
Caspofungin	2 (40)	0
Total	***5(100)***	***2 (100)***

Supplementary S2. Defined daily doses during preliminary and developed use of ASP

Preliminary ASP (n=81)					Developed ASP (n=105)				
**Antimicrobial**	**Route**	**Location of infection**	**DDD**	**Total cost QAR (USD)**	**Antimicrobial**	**Route**	**Location of infection**	**DDD**	**Total cost QAR (USD)**
Amikacin	Parenteral	N.R	1000	1,682.6 (462.25)	Amikacin	Parenteral	Pus culture	1000	286.4 (78.68)
Amikacin	Parenteral	Blood	1000	13,031.2 (3580)	Amikacin	Parenteral	Urine culture	1000	179 (49.18)
Amphotericin B	Parenteral	Blood	35	5,173.3 (1421.2)	Amikacin	Parenteral	Blood	1000	71.6 (19.67)
Amphotericin B	Parenteral	N.R	35	22,848.7 (6277.1)	Amphotericin	Parenteral	N.R	35	15519.9 (4263.71)
Anidulafungin	Parenteral	Blood	100	56,434.4 (15504)	Amphotericin	Parenteral	N.R (every other day dosing	35	25866.5 (7106.18)
Anidulafungin	Parenteral	Pus	100	67,993.2 (18679.45)	Anidulafungin	Parenteral	Wound culture	100	14278.6 (3922.69)
Caspofungin	Parenteral	Blood	50	815,917.2 (224153.08)	Caspofungin	Parenteral	Urine	50	69935.8 (19213.13)
Caspofungin	Parenteral	N.R	50	60,611.0 (16651.37)	Caspofungin	Parenteral	Ova and paracites	50	60611 (16651.37)
Caspofungin	Parenteral	Mouth	50	36,133.5 (9926.79)	Caspofungin	Parenteral	Blood	50	6993.6 (1921.32)

Ciprofloxacin	Parenteral	N.R	800	3,347.8 (919.73)	Cefazolin	Parenteral	N.R	3000	27.8 (7.64)
Ciprofloxacin	Parenteral	Blood	800	4,892.9 (1344.20)	Cefazolin	Parenteral	Stool	3000	44.4 (12.2)
Fluconazole	Parenteral	Abdomen	200	2,546.9 (699.70)	Cefepime	Parenteral	Blood	4000	257.1 (70.63)
Fluconazole	Oral	N.R	200	1,019.7 (280.14)	Cefepime	Parenteral	Urine culture	4000	321.3 (88.27)
Fluconazole	Parenteral	N.R	200	1,833.8 (503.79)	Cefepime	Parenteral	N.R	4000	514.1 (141.24)
Fluconazole	Oral	Urine	200	144.0 (39.56)	Ciprofloxacin	Parenteral	N.R	800	6051.7 (1662.55)
Fluconazole	Parenteral	Acid-fast bacilli smear	200	203.8 (55.99)	Ciprofloxacin	Parenteral	Blood and urine	800	128.8 (35.38)
Fluconazole	Oral	Stool	200	36.0 (9.89)	Ciprofloxacin	Parenteral	Urine culture	800	4120.3 (1131.95)
Fluconazole	Oral	Stomach and pyloric canal	200	76.0 (20.88)	Ciprofloxacin	Parenteral	Clostridioides difficile	800	386.3 (106.13)
Fluconazole	Parenteral	Nose	200	331.1 (90.96)	Ciprofloxacin	Parenteral	Pus culture	800	901.3 (247.61)
Fluconazole	Parenteral	Blood	200	840.5 (230.91)	Ciprofloxacin	Parenteral	Stool	800	1158.8 (318.35)

Fluconazole	Parenteral	Intra-abdomenal	200	2,546.9 (699.70)	Ciprofloxacin	Parenteral	Blood	800	643.8 (176.87)
Fluconazole	Oral	Blood	200	807.8 (221.92)	Ciprofloxacin	Oral	N.R	1000	9.8 (2.69)
Moxifloxacin	Parenteral	N.R	400	440.1 (120.91)	Ertapenem	Parenteral	Urine culture	1000	14277.4 (3922.36)
Tigecycline	Parenteral	Blood	100	41,185.9 (11314.81)	Ertapenem	Parenteral	Blood	1000	17763.8 (4880.16)
Voriconazole	Oral	Blood	400	6,136.3 (1685.80)	Ertapenem	Parenteral	Pus culture	1000	498 (136.81)
Voriconazole	Oral	Wound	400	955.9 (262.61)	Ertapenem	Parenteral	Sputum	1000	3320.3 (912.17)
Voriconazole	Parenteral	Blood	400	9,916.6 (2724.34)	Ertapenem	Parenteral	Clostridioides difficile	1000	1992.2 (547.31)
Voriconazole	Oral	Peripherally Inserted Central Catheter tube site	400	10,175.8 (2795.55)	Ertapenem	Parenteral	Blood and urine	1000	3984.4 (1094.62)
Posaconazole	Oral	N.R	300	212,464.6 (58369.40)	Ertapenem	Parenteral	Wound culture	1000	5312.5 (1459.48)
Posaconazole	Oral	Blood	300	153,216.0 (42092.31)	Ertapenem	Parenteral	N.R	1000	3154.3 (866.57)

Posaconazole	Oral	Catheter tip	300	2,641.7 (725.74)	Fluconazole	Parenteral	Urine	200	534.8 (146.92)
Posaconazole	Oral	Neck	300	14,340.4 (3939.67)	Fluconazole	Parenteral	Pus culture	200	662.2 (181.92)
Posaconazole	Oral	Acid-fast bacilli smear	300	1,509.5 (414.70)	Linezolid	Parenteral	Blood	1200	729.3 (200.36)
Posaconazole	Oral	Tonsil	300	17,359.5 (4769.09)	Linezolid	Parenteral	Wound culture	1200	72.9 (20.03)
Levofloxacin	Oral	N.R	500	74.5 (20.47)	Moxifloxacin	Oral	N.R	400	23 (6.32)
Ertapenem	Parenteral	Blood	1000	830.1 (228.05)	Moxifloxacin	Oral	Blood	400	23 (6.32)
					Moxifloxacin	Parenteral	Sputum	400	528.1 (145.08)
					Moxifloxacin	Parenteral	Blood and stool	400	264.1 (72.55)
					Moxifloxacin	Parenteral	Blood	400	2200.5 (604.53)
					Moxifloxacin	Parenteral	Blood and urine	400	352.1 (96.73)
					Moxifloxacin	Parenteral	Clostridioides difficile	400	176 (48.35)
					Moxifloxacin	Parenteral	N.R	400	704.2 (193.46)
					Teicoplanin	Parenteral	Pus culture	400	1589.7 (436.73)
					Teicoplanin	Parenteral	Blood and urine	400	132.5 (36.4)

					Tigecycline	Parenteral	Pus culture	100	8998.6 (2472.14)
					Tigecycline	Parenteral	Body fluid	100	2076.6 (570.49)
					Posaconazol	Oral	Ova and paracites	300	22642.8 (6220.55)
Total			***11,620***	**1,569,699 (USD 431,236)**<br><br>19,379 (USD 5,309) per patient	***Total***			***42,920***	**300,321 (USD 82,505.7)**<br><br>2,860 (USD 784) per patient

*ASP: antimicrobial stewardship program; DDD: defined daily dose; N.R: not reported

Supplementary S3. Sensitivity analyses and their uncertainty distributions

One-way sensitivity analysis							
**Variable**	**Distribution**	**Preliminary ASP**		**Developed ASP**		**Outcomes**	
		**Point estimate,**	**Variation range**	**Point estimate**	**Variation range**	**Reduced cost of resource use (total), 95% CI, QAR (USD)**	**Reduced cost of resource use (per patient), 95% CI, QAR (USD)**
Cost of hospitalization, QAR (USD)	Triangular	6,106 (1,677.13)	5,190 (1,425.53), 7,021 (1,928.45)	6,106 (1,677.13)	5,190 (1,425.53), 7,021 (,1928.45)	10,574,476 (797,769.08), 15,570,233 (4,276,655.66)	154,003 (42,192), 189,812 (52,003)
Length of hospital stay (Days, per 100-patient beds)	Triangular	2756	2343, 3169	748	636, 860	10,808,083 (2,961,118), 15,625,835 (4,281,051)	165,942 (45,464), 196,039 (53,709)
Additional hospital stay due to ADEs (days)	Triangular	1 for non-injectable medications and 2 for injectable medications	1.6, 2.4 (non-injectables)<br><br>0.8, 1.2 (injectables)	1 for non-injectable medications and 2 for injectable medications	1.6, 2.4 (non-injectables)<br><br>0.8, 1.2 (injectables)	13,177,805 (3,610,357), 13,247,112 (3,629,346)	159,206 (43,618), 185,827 (50,912)
**Multivariate sensitivity analysis**							
**Variable**	**Distribution**	**Point estimate**	**Variation range**	**Point estimate**	**Variation range**	**Reduced cost of resource use (total), 95% CI, QAR (USD))**	**Reduced cost of resource use (per patient), 95% CI, QAR (USD)**

Cost of hospitalization QAR (USD)	Triangular	6,106 (1677.13)	5,190 (1,425.53), 7,021 (1,928.45)	6,106 (1,677.13)	5,190 (1425.53), 7,021 (1,928.45)	9,601,786 (2,637,310.08), 16,674,087 (4,579,849.80)	161,700 (44,301), 197,649 (54,150)
Length of hospital stay (Days)	Triangular	2,756	2343, 3169	748	636, 860		
Additional hospital stay due to ADEs (days)	Triangular	1 for non-injectable medications and 2 for injectable medications	1.6, 2.4 (non-injectables)<br><br>0.8, 1.2 (injectables)	1 for non-injectable medications and 2 for injectable medications	1.6, 2.4 (non-injectables)<br><br>0.8, 1.2 (injectables)		
ASP: antimicrobial stewardship program; QAR: Qatari Riyals; USD: United States Dollar; ADE: adverse drug events							

Supplementary S4. Probability of reduction in cost of resource use in favor of developed ASP, one-way sensitivity analysis

A - Net benefit in length of hospital stay

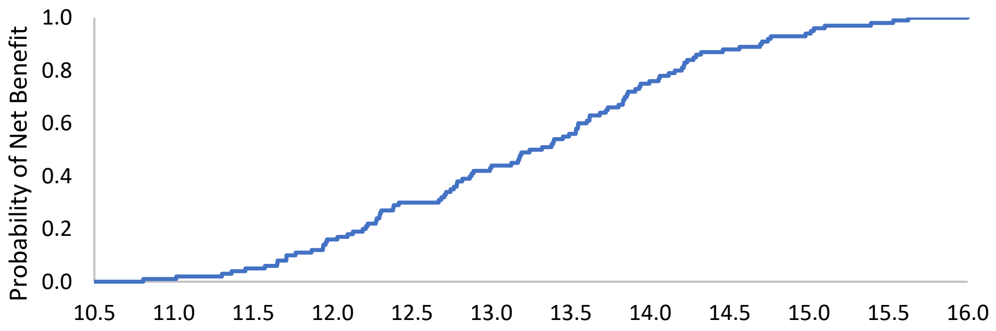

Net Benefit (length of hospital stay) (Qatari Riyal (Million), QAR). 1 USD= 3.65 QAR

B - Net Benefit in additional hospital stay due to adverse drug events

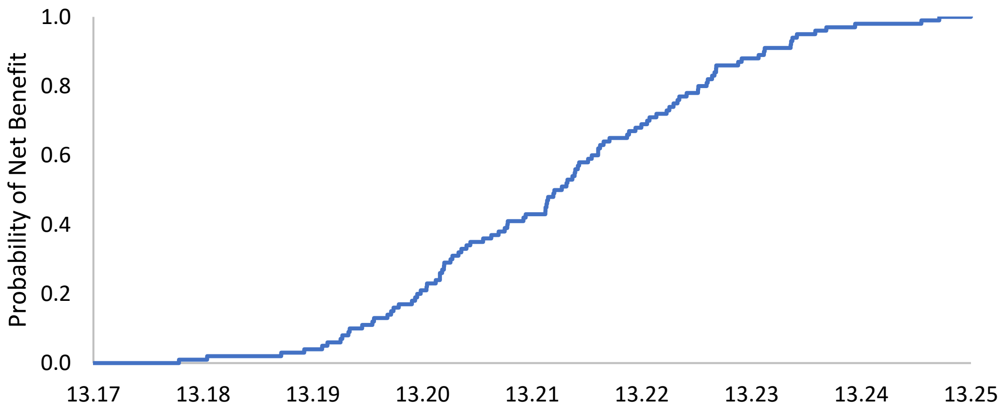

Net Benefit (additional hospital stay due to ADEs) (Qatari Riyal (Million), QAR). 1 USD= 3.65 QAR

C - Net Benefit in hospitalization cost

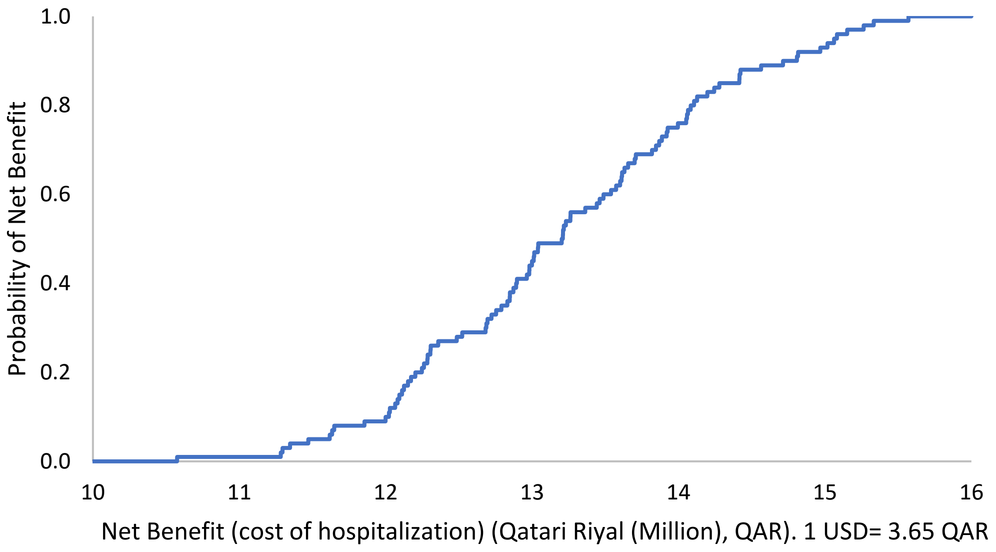

Supplementary S5. Reduction in cost of resource use in favor of developed ASP, multivariate sensitivity analysis

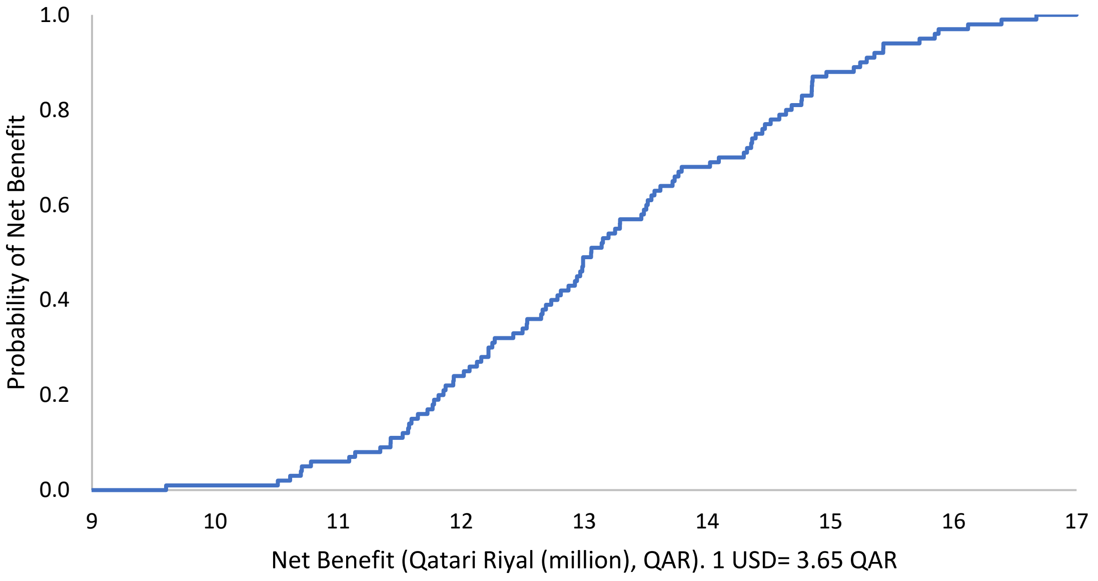

Supplement: Supplementary file 1 [file antibiotics-14-01204-s001.zip › antibiotics-3911879-supplementary.pdf]
